# A randomized clinical trial to evaluate the efficacy of L-carnitine L-tartrate to modulate the effects of SARS-CoV-2 infection

**DOI:** 10.3389/fnut.2023.1134162

**Published:** 2023-07-20

**Authors:** Roberto Badaro, Josiane Dantas Viana Barbosa, Cesar Augusto de Araujo Neto, Bruna Aparecida Souza Machado, Milena Botelho Pereira Soares, Valter de Senna, Marcelo Taddeo, Lila Teixeira de Araújo, Shane Durkee, Raymond Donninger, Kevin Judge, Zainulabedin Saiyed

**Affiliations:** ^1^Institute of Health Technologies (ITS), University Center SENAI/CIMATEC, Salvador, Bahia, Brazil; ^2^Image Diagnosis, Salvador, Bahia, Brazil; ^3^Gonçalo Moniz Institute, FIOCRUZ, Salvador, Bahia, Brazil; ^4^Federal University of Bahia, Salvador, Bahia, Brazil; ^5^Research and Development, Lonza Greenwood, LLC, Greenwood, SC, United States; ^6^Research and Development, Lonza Biologics, Slough, United Kingdom

**Keywords:** L-carnitine, COVID-19, SARS-CoV-2, pneumonia, infection

## Abstract

**Introduction:**

L-carnitine (LC) has been associated with inflammatory mediator reduction and with downregulating the angiotensin-converting enzyme-2 (ACE2) receptor, which is the target of SARS-CoV-2 attachment.

**Methods:**

This pilot phase 2 randomized, double-blind placebo-controlled trial contained two cohorts. Cohort 1 comprised 101 individuals with negative RT-PCR SARS-CoV-2 test results who cohabitated with an individual diagnosed with SARS-CoV-2 infection. Cohort 2 comprised 122 individuals with positive SARS-CoV-2 RT-PCR test results who were asymptomatic or had mild COVID-19 pneumonia symptoms. Participants in each cohort were randomized 1:1 to receive either 2 g elemental oral LC supplementation or placebo daily for 21 days. Primary endpoints included adverse events, SARS-CoV-2 infection incidence in Cohort 1, and disease progressions in Cohort 2. Secondary endpoints included between-group laboratory profile comparisons and Cohort 2 ACE1/ACE2 plasma levels. Disease progression was compared between the Cohort 2 groups using chest computed tomography.

**Results:**

In Cohort 1, two SARS-CoV-2 infections occurred in each group. The common adverse events included headache, dyspnea, and tiredness. In Cohort 2, platelet counts were elevated, and fibrinogen levels reduced in the LC group compared with those of the placebo group.

**Conclusion:**

Our study showed that LC was well-tolerated and suggests it modulates coagulation pathways. Furthermore, chest computed tomography images of the Cohort 2 LC group showed significant lung lesion improvement, suggesting that LC may slow COVID-19 progression.

## 1. Introduction

L-carnitine (LC) is a molecule that transports long-chain fatty acids into the mitochondria for fat oxidation and is derived mostly from dietary intake (1–3). A prior study has suggested that LC is involved in inflammatory pathway mediation by reducing inflammatory cytokines (4–6). By reducing inflammation, oxidative stress, and myocyte necrosis, LC may thus provide cardioprotective effects (7–10).

Such effects may be of particular interest concerning COVID-19 treatment (11). In addition to respiratory and metabolic dysfunction, patients with COVID-19 can experience inflammatory cytokine storms leading to organ failure (12, 13). These exacerbated inflammatory responses entail upregulation of the angiotensin-converting enzyme-2 (ACE2) receptor, to which the SARS-CoV-2 virus spike protein (S protein) binds, thereby gaining entry to host cells (11, 13). This binding also relies upon transmembrane serine protease 2 (TMPRSS2) and furin peptidase, which mediate the cleavage of the spike protein at the S2’ site and S1/2 sites, respectively (14, 15).

Because of its role in inflammation mediation, LC has been studied for relationships with ACE2 and viral infection, with some evidence suggesting that LC inhibits hepatitis C virus propagation and has antioxidant activity (16, 17). In our previous research, we found a dose-dependent reduction in SARS-2-CoV infection of human lung epithelial cells following treatment with LC. The same study showed that LC supplementation in humans was associated with decreased ACE2. Furthermore, we showed that LC supplementation was associated with decreased TMPRSS2 and furin (16).

Hence, our objective in this study was to determine the safety of daily LC supplementation in individuals who are at risk of SARS-CoV-2 infection or are already SARS-CoV-2 positive, and to evaluate whether daily LC supplementation is protective against SARS-CoV-2 infection or slows the progression of COVID-19.

## 2. Materials and methods

We conducted a double-blind, two-cohort, parallel design, placebo-controlled study to evaluate the safety and efficacy of investigational LC (Carnipure® tartrate), in its salt form, L carnitine L-tartrate (LCLT), in adults at risk of SARS-CoV-2-infection (Cohort 1) or disease progression (Cohort 2) in patients with SARS-CoV-2 infection.

### 2.1. Study participants

The study started in March, and the first volunteer was included in March 24, 2021, and the last volunteer was included in September 17, 2021. Participants were recruited from research centers located in Salvador, Bahia, Valinhos, and Bernardo do Campo, São Paulo, Brazil. Co-participating centers involved in recruitment and participant follow-up included Azidus Brazil in Valinhos and CEMEC in Sao Bernardo do Campo. Of the 1,283 screened individuals, 223 men and women were enrolled, ranging in age from 18 to 85 years. The study comprised two cohorts: Cohort 1 included participants with negative SARS-CoV-2 RT-PCR test results on day 1, and Cohort 2 included participants with positive SARS-CoV-2 RT-PCR test results. Participants provided written informed consent to the study prior to any study procedures. The Institutional Review Board (IRB) and Independent Ethics Committee (IEC) approved the study and the informed consent form (CAAE: 42972521.9.0000.9287). All participants signed the informed consent form. The study was registered at https://www.clinicaltrials.gov (NCT05446961) and was conducted in accordance with Good Clinical Practice Guidelines (GCP) and Brazilian National Health Council resolution 466/2021.

Participants in Cohort 1 had reported a history of cohabitation with a confirmed family member, friend, or cohabitant who had received a diagnosis of SARS-CoV-2 infection based on a positive RT-PCR result in the previous 7 days. Cohort 1 participants received a SARS-CoV-2 negative result on screening immediately after contact with the infected cohabitant and had a negative RT-PCR and serological result prior to study initiation. Participants in Cohort 2 had a medical history and physical exam findings consistent with asymptomatic or mild COVID-19 pneumonia confirmed with virology test results.

Excluded individuals had received any SARS-CoV-2 vaccine or did not have virology confirmation through the laboratory of SENAI CIMATEC. Women who were pregnant, lactating, or had not adhered to adequate contraception from at least 30 days prior to the study entry and did not plan to for ≤1 month after the final supplementation were also excluded. Patients with severe COVID-19 pneumonia, according to the CDC criteria (SpO2 < 94% on room air, a ratio of arterial partial pressure of oxygen to fraction inspired oxygen of <300 mm Hg, respiratory rate of >30 breaths/min, or lung infiltrates >50%) and who were critically ill with respiratory failure, septic shock, and multiple organ failure were also excluded (18). Individuals with a positive screening test result for hepatitis B surface antigen, hepatitis C virus antibody, or HIV types 1 or 2 antibodies were excluded. Individuals with a history of pulmonary, neurologic, hepatic, rheumatic, hematologic, or renal chronic disease, who were treated with immunosuppressive therapy in the 6 months prior to screening, or who used medication or supplements that may have confounded the study results were also excluded.

**Figure 1:**
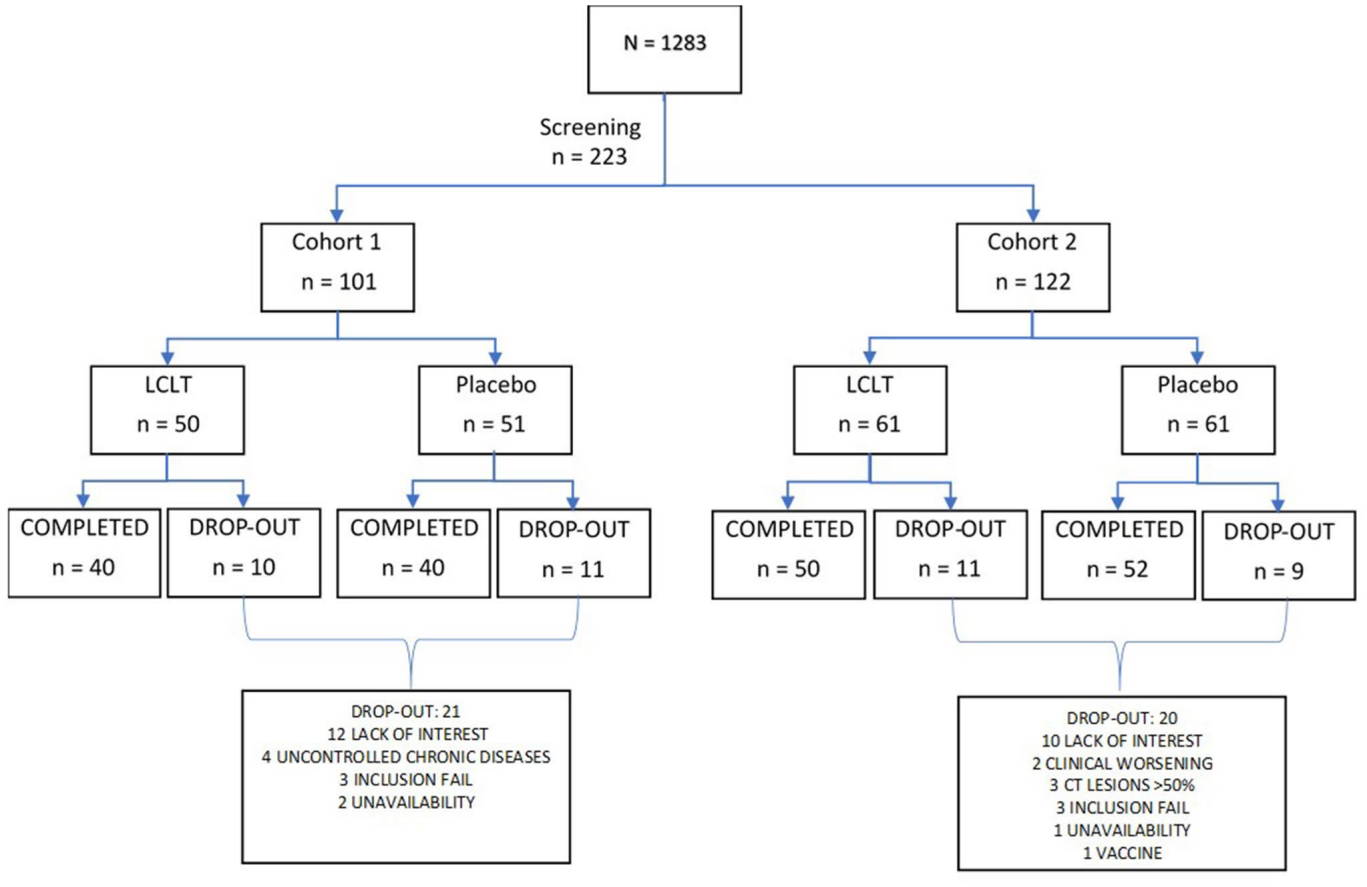
Participants randomization in both cohorts. LCLT, L-carnitine L-tartrate.

Participant screening entailed an interview-based medical history, review of concomitant medications or supplements, and physical examination with vital signs. Laboratory evaluation included a nasopharyngeal swab for RT-PCR detection of SARS-CoV-2 at participant screening. Blood serum chemistry, hematology, and CRP levels were also obtained. After their eligibility was confirmed, the participants in both cohorts were then randomized 1:1 to receive LCLT supplement (Group A) or placebo (Group B) for 21 days in balanced blocks (Figure 1). Follow-up of participants continued for 28 days. The randomization was scheduled using SAS programming code. The randomization schedule remained blinded to the sponsor, participants, investigator, and site staff, from the time of participant screening to the final data lock.

### 2.2. Supplementation

The LCLT was supplied by Lonza Greenwood, SC and is composed of 68% elemental L-carnitine and 32% tartaric acid, which can be taken safely, per the European Food Safety Authority (EFSA) at up to 3 g (19). Each 3 g dose of LCLT delivers 2 g of elemental L-carnitine. Both the supplement and placebo were manufactured in capsule format with maltodextrin replacing the LCLT in the placebo capsules. The participants received an amount that was enough for 1 week of supplement consumption. They received instructions to swallow it **three** times a day (**three** capsules of 1 g each), after main meals, recording daily the quantity of capsules ingested on a specific form named “Participant’s daily card.” For each return visit to the site of research, aiming to receive, the participants answered to an adherence form, filled out by the pharmacist, immediately after medical appointment. Then participants received a new amount of the investigational product.

### 2.3. Study procedure

All participants underwent clinical evaluation and blood sample collection on day 1 and were provided a 7-day supply of supplement or placebo capsules with instructions on their use. Empty dose containers were exchanged for new 7-day supplies on days 7 and 14, with empty dose containers also collected on day 21. Blood samples were collected from participants on days 7, 14, and 21. Clinical evaluations of participants were repeated on days 7, 14, and 21. Participants in Cohort 2 underwent high-resolution chest CT on days 1, 7, 14, and 21 to monitor pneumonia severity. Also, for Cohort 2, specific serum markers including ACE1 and ACE2, were analyzed on days 1 and 21.

Diary cards were provided to participants on days 1, 7, 14, and 21, onto which participants were instructed to record their temperature, adverse events (AEs), medication, and supplementation. Completed cards were returned and collected by the investigator at each visit. The investigator collected, verified completed diary cards, and qualified staff interviewed participants to assess the occurrence of unsolicited AEs, serious adverse events (SAEs), and adverse events of special interest (AES). Participants were instructed to contact the study investigator immediately if they developed uncommon signs or symptoms or had any medical condition resulting in hospitalization or an emergency room visit.

### 2.4. Chest high resolution computer tomography scanning protocol

High resolution computer tomography (HRCT) was performed in both study cohorts on days 1, 7, 14, and 21. The test protocol for evaluation of suspected COVID-19 was based on high resolution volumetric scanning of the chest, with special attention to the selection of parameters ensuring creation of motion-free images and adequate image quality at a reduced radiation dose. The optimal chest HRCT scan for COVID-19 characterization had to be a non-contrast examination, except in context of acute respiratory decline, in which case CT angiography was justified to detect acute pulmonary embolisms.

**Figure 2:**
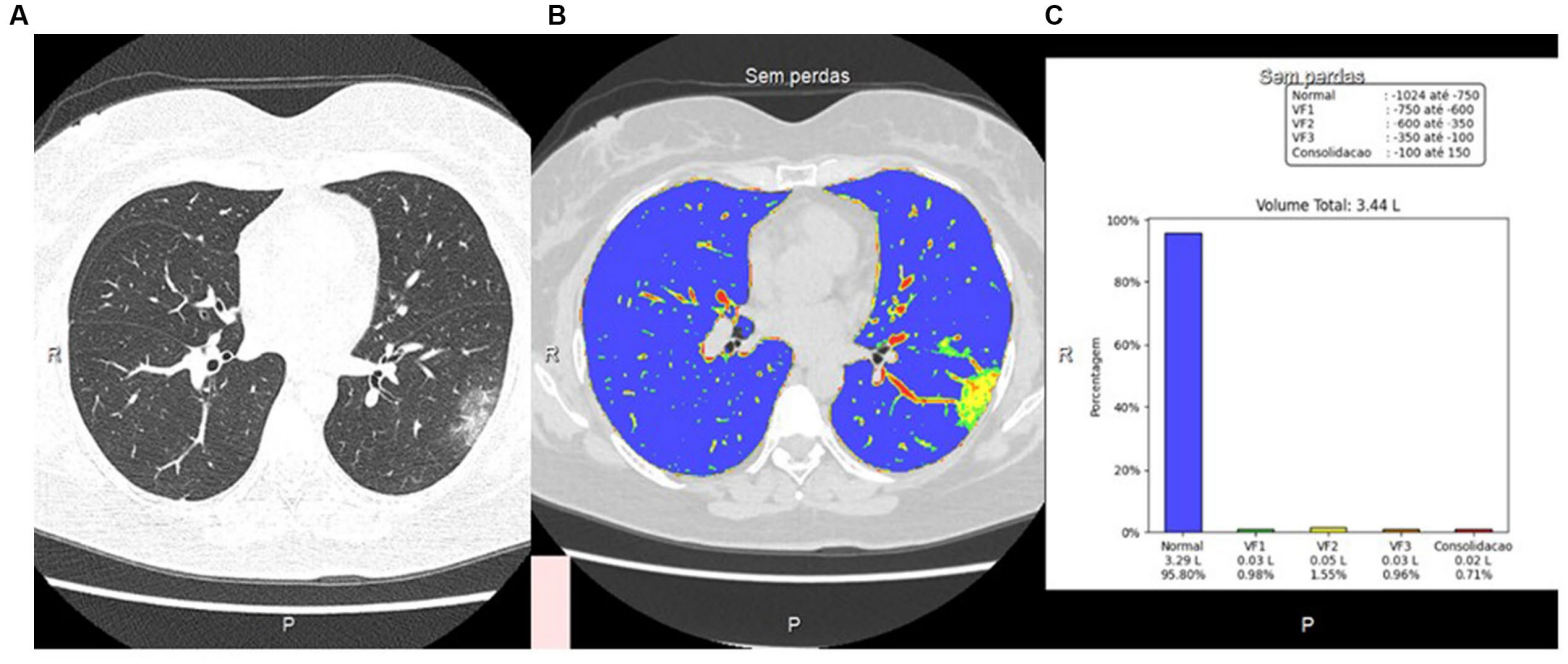
CT image using visual and artificial intelligence (AI) analysis. **(A)** Axial HRCT scan using low-dose radiation technique; **(B)** Quantification of pulmonary findings with the deep reading system; **(C)** Graphical representation of quantitative analysis with the deep learning system.

The characteristic feature or COVID-19 pneumonia is the presence of ground glass lesions in the lung. In here, we estimated the percentage of ground glass lesions with or without consolidation by applying a classification criteria scale as: (1) less than 25%; (2) between 25 and 50%; and (3) more than 50%. In addition, we also employed an Artificial Intelligence (AI) analysis using an automatized deep learning classification to get a more accurate estimate on the percentage of lung area involved in lesions. Figure 2 shows a representative image that was analyzed using the AI analysis tool.

### 2.5. End points

The primary end points included the occurrence of solicited AEs during the supplementation period and unsolicited AEs within the 28 days following the start of supplementation. Primary end points for Cohort 1 included hematologic, renal, liver, and other chemistry, mineral, and electrolyte abnormalities and the incidence of new SARS-CoV-2 infections in the supplement group compared with that in the placebo group. For Cohort 2, the primary end points consisted of the number of cases of SARS-CoV-2 infections that progressed from asymptomatic or mild COVID-19 to severe pneumonia in the supplement and placebo groups. Progression of disease in Cohort 2 was defined as the persistence of fever for >48 h after trial initiation, increased respiratory frequency after trial initiation, muscle weakness, chest pain/worsening chest pain, cough/worsening cough, or headache/worsening headache 48 h after the trial initiation, or loss of appetite/worsening appetite loss, or diarrhea/worsening diarrhea in the week after trial initiation, or shortness of breath. Primary end points also included hospitalization and oxygen requirements, with severity parameters defined by CDC pneumonia criteria (18).

Study secondary end points included the comparison of ACE1 and ACE2 receptor levels between the supplement and placebo groups of Cohort 2 (analyzed at baseline and every 7 days for 21 days) and the ACE1/ACE2 ratios in both supplement and placebo groups of each cohort.

Secondary end points also included laboratory-based evaluation of disease severity or progression in Cohort 1 (if the participant contracted SARS-CoV2 infection) and the comparison of CRP levels at study enrollment and at days 1, 7, 14, and 21 between the supplement and placebo groups in both cohorts. The comparison of basic laboratory profiles between supplement and placebo groups in each cohort at study enrollment and days 7, 14, and 21 were included in the secondary end points. Lastly, comparative disease progression as observed on lung chest CT scan images between the supplement and placebo groups of Cohort 2 was included as a secondary end point. The safety end point included participant-reported AEs.

### 2.6. Statistical analysis

We used three different statistical tests to compare different study parameters: the Wilcoxon test, the Kruskal-Wallis test, and the Friedman test. The Kruskal-Wallis test is a nonparametric statistical test used to assess the equality among the distributions associated with the participant visits. The Wilcoxon rank-sum test was used to determine the pairwise statistical difference in mean changes among the study groups. On the other hand, the Wilcoxon signed-rank test is a nonparametric test used for changes between study visits within the same group. Finally, the Friedman test is a nonparametric analog of the ANOVA test. In this study, it was used to assess whether the treatment effect was the same between the two groups.

## 3. Results

Of the 1,283 screened individuals, 223 were enrolled in one of the two cohorts: Cohort 1 included 101 (45%) individuals with negative RT-PCR results who cohabited with individuals infected with SARS-CoV-2, and Cohort 2 included 122 (55%) recently infected individuals who were asymptomatic or had mild pneumonia. Of the 223 enrolled participants, 95 (43%) were men, and 128 (57%) were women. Within Cohort 1, there were 43 men and 58 women, and in Cohort 2, there were 52 men and 70 women.

### 3.1. Safety

Prior to randomization, one patient (of 224 enrolled participants) died, with no relationship between the death and the study or the supplement identified by the CIMATEC committee, since the death occurred prior to collect blood samples or consume the investigational supplement. Of the 223 participants, 182 completed the 21 days of LCLT or placebo supplementation. The LCLT supplement was well-tolerated by the participants. The most common AEs reported for both cohorts included headache responsive to analgesia, tiredness, and dyspnea (Table 1). There were no significant differences in the number of reported AEs between the LCLT and placebo groups in either cohort. No SAEs were reported among either LCLT or placebo groups.

**Table 1:** Adverse events in Cohort 1 (*n* = 101) and Cohort 2 (*n* = 122) combined.

Adverse events (*N*)	Follow-up	Coorte 1			Coorte 2		
		LCLT	Placebo	*p* value	LCLT	Placebo	*p* value
		*n*1 = 51	*n*2 = 50	*p*1 < *p*2	*n*1 = 61	*n*2 = 61	*p*1 < *p*2
**Headache**	D1	4	5	0.512	20	29	0.930
	D7	4	5	0.512	12	20	0.925
	D14	3	8	0.905	16	8	0.055
	D21	0	2	0.767	6	8	0.612
**Dyspnea**	D1	2	2	0.500	2	10	0.983
	D7	0	1	0.504	1	3	0.694
	D14	1	1	0.500	5	3	0.357
	D21	1	1	0.500	3	3	0.500
**Tiredness**	D1	3	3	0.500	29	32	0.641
	D7	0	3	0.883	14	21	0.885
	D14	1	1	0.500	12	12	0.500
	D21	1	2	0.507	7	10	0.700

LCLT, L carnitine L-tartrate.

### 3.2. Cohort 1

During the 21-day follow-up period, there were only four cases of SARS-CoV-2 infection in Cohort 1, evenly distributed between the LCLT (*n* = 2) and placebo (*n* = 2) groups and, therefore, the majority of the participants remained free of COVID-19 during the follow-up period. Thus, the primary end point of new SARS-CoV-2 infection in supplemented group compared to placebo was not statistically significant. In addition, hematologic and biochemical analyses, including ferritin and fibrinogen, performed during the follow-up period, did not show any significant differences between the supplement group compared with the placebo group. The CRP levels were significantly higher in the placebo group compared to LCLT group at day 21 (Table 2).

**Table 2:** Biochemical and hematological analyses of Cohort 1.

Endpoint	D01				D21			
	Range	LCLT* group (*N* = 51)	Placebo* group (*N* = 50)	*p* value Mann–Whitney	Range	LCLT* group (*N* = 42)	Placebo*group (*N* = 41)	*p* value Mann–Whitney
Basophils (μL)	0–109	23.57 ± 17.09	27.36 ± 22.53	0.45	0–111	25.56 ± 19.99	25.85 ± 20.54	0.93
Eosinophils (μL)	0–1,557	206.27 ± 246.07	193.3 ± 187.31	0.98	5–874	197.36 ± 125.39	193.67 ± 190.37	0.18
Ferritin (ng/mL)	2.9–1117.5	175.37 ± 148.11	199.9 ± 216.38	0.86	5–808.3	164.63 ± 158.23	159.72 ± 162.44	0.69
Fibrinogen (mg/dL)	214–509	307.27 ± 53.4	315.48 ± 65.01	0.53	200–522	303.68 ± 48.19	322.59 ± 71.4	0.43
Lymphocytes (μL)	789–3,883	1995.55 ± 471.35	2026.72 ± 636.87	0.97	849–3,315	1926.13 ± 392.69	1893.44 ± 585.83	0.60
Monocytes (μL)	71–934	370.1 ± 132.01	336.22 ± 104.84	0.26	57–828	374.62 ± 127.74	333.77 ± 115.18	0.20
Platelets (μL.10^−3^)	95–609	256.31 ± 58.01	273.78 ± 84	0.27	75–419	263.13 ± 66.02	284.41 ± 66.75	0.09
CRP (mg/dL)	0.03–15	1.50 ± 1.55	2.05 ± 3.03	0.26	0.03–15	1.48 ± 1.28	2.96 ± 3.93	0.02

LCLT, L carnitine L-tartrate; ^*^Values represent the mean ± SD.

### 3.3. Cohort 2

**Figure 3:**
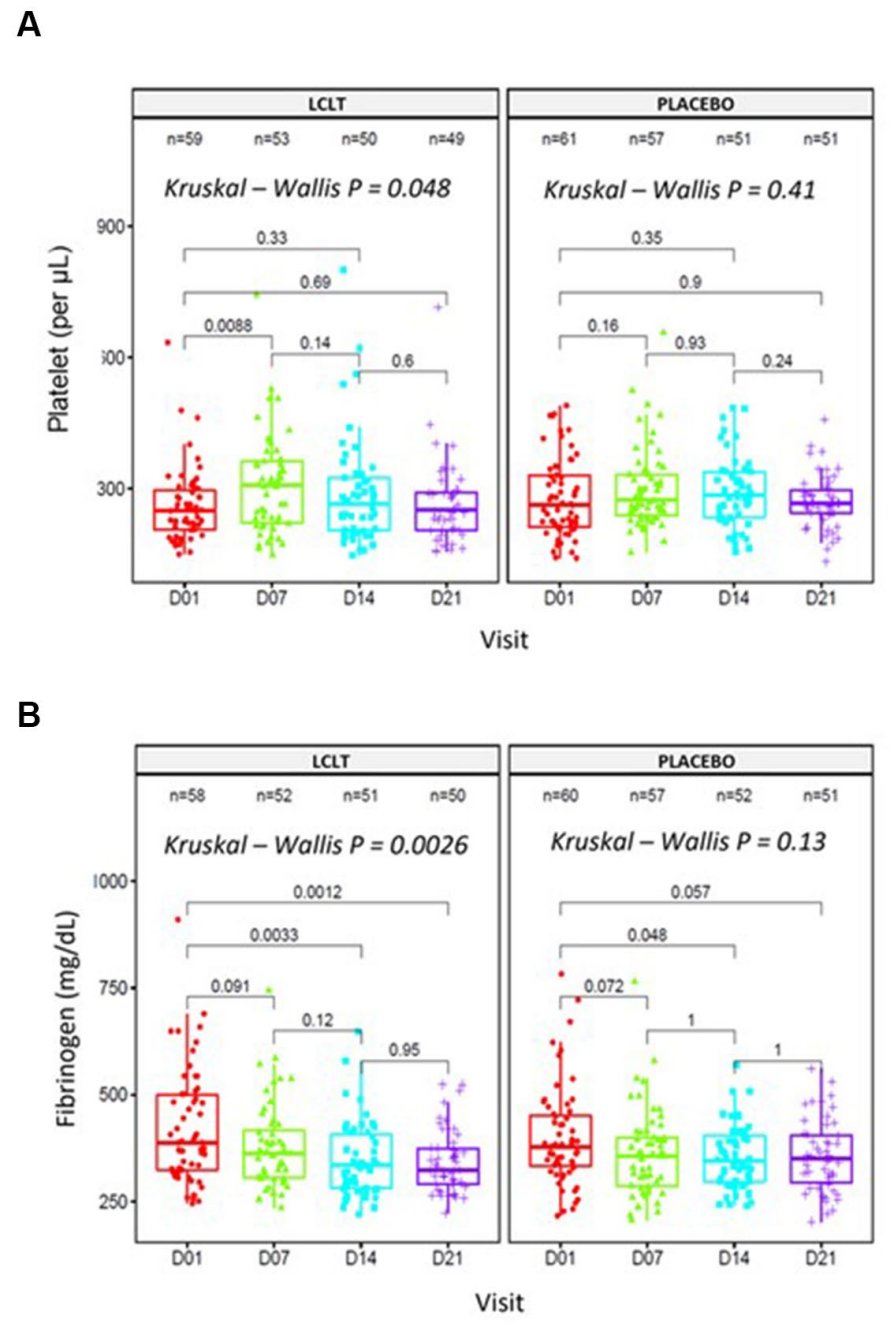
Platelet counts and fibrinogen in entire cohort 2. Analyses performed in blood samples collected at days 01, 07, 14, and 21. **(A)** Platelet counts [Friedman test, *X*^2^(1) = 4, *p* = 0.046, *n* = 4]. **(B)** Fibrinogen levels [Friedman test, *X*^2^(1) = 1, *p* = 0.32, *n* = 4].

In Cohort 2, no significant hematologic parameter alterations were observed in the leukocyte counts for either group. However, the platelet count was higher in the LCLT group than in the placebo group (*p* = 0.048; Figure 3A). For most participants, the platelet numbers fell within the normal reference range (140,000–500,000/μL.10^−3^). In the LCLT group, the platelet levels increased significantly from day 1 to 7 (*p* = 0.009; Table 3) and were then followed by a decrease in levels observed from days 7 to 21. The distributions and medians of fibrinogen levels in the LCLT group on days 1, 7, 14, and 21 (particularly between days 1 and 14) were significantly different (*p* = 0.003). The Kruskal-Wallis test showed that the distributions in the LCLT group differed (*p* = 0.003; Figure 3B). A similar difference was observed between days 1 and 21 (*p* = 0.001) in the LCLT group. This pattern was not observed in the placebo group’s fibrinogen levels. On day 1, most of the participants had elevated (> 400 mg/dL) fibrinogen levels. The fibrinogen levels decreased significantly from day 1 to 14 in both the LCLT and placebo groups (*p* = 0.003, *p* = 0.048, respectively). A significant decrease in fibrinogen also occurred in the LCLT group between days 1 and 21 (*p* = 0.001). There was a similar trend of fibrinogen level decrease from day 1 to 21 in the placebo group (*p* = 0.057). Similarly, there were no changes in or differences between the LCLT or placebo groups of Cohort 2 in blood sodium, potassium, ALT, AST, total bilirubin, ALP, creatinine, fasting glucose, D-dimer, and ACE2 levels during the study (data not shown). The serum ACE1 levels were increased in the LCLT and the placebo groups between days 1 and 21, however, this change reached statistical significance only for the LCLT group (*p* = 0.029; Table 3). The chest CT scan findings in Cohort 2 participants did not show significant differences between the LCLT and placebo groups following either non-adjusted or adjusted CT scan analysis. For all other laboratory parameters, such as hematologic, renal urea, creatinine, liver enzymes SGOT, SGTP, alkaline phosphatase, lactate dehydrogenase (LDH), total bilirubin, as well as Na, K, Mg, Zinc, and fasting glucose, there was no difference between placebo and LCLT groups. Moreover, the comparison of CRP levels at the different timepoints evaluated did not show any significant differences between the LCLT and placebo groups, as presented in Table 3.

**Table 3:** Within-group and between-group hanges observed in Cohort 2.

Endpoint per group	Day	Difference median^*^ (95% CI)	Significant change in LCLT group (*p* value)	Significant change in Placebo group (*p* value)	LCLT vs. Placebo significant difference (*p* value)
Platelets (μL.10^−3^) in total group (*n* = 122)	1 vs. 7	11.5 (7.0; 20.5)	Yes (0.009)	No (0.16)	No (0.56)
	1 vs. 14	7.5 (−1.5; 13.5)	No (0.33)	No (0.35)	No (0.71)
	1 vs. 21	3.0 (−2.5; 11.0)	No (0.69)	No (0.91)	No (0.62)
Platelets (μL.10^−3^) in subgroup (*n* = 23)	1 vs. 7	91.0 (53.5; 141)	Yes (0.006)	Yes (0.018)	No (0.71)
	1 vs. 14	101.0 (41.5; 163.5)	Yes (0.018)	Yes (0.013)	No (0.68)
	1 vs. 21	33.0 (10; 67.5)	No (0.23)	No (0.083)	No (0.54)
Fibrinogen (mg/dL) in total group (*n* = 122)	1 vs. 7	−8.0 (−19.5; −3.5)	No (0.091)	No (0.072)	No (0.95)
	1 vs. 14	−19.0 (−31.5; −11.5)	Yes (0.003)	Yes (0.048)	No (0.22)
	1 vs. 21	−20.5 (−34.5; −13.5)	Yes (0.001)	No (0.057)	No (0.30)
Fibrinogen (mg/dL) in subgroup (*n* = 23)	1 vs. 7	−3.5 (−51.0; 57.5)	No (0.88)	No (0.88)	No (0.79)
	1 vs. 14	−39.5 (−110.5; 10.0)	Yes (0.038)	No (0.83)	No (0.13)
	1 vs. 21	−47.0 (−96.0; −9.5)	No (0.12)	No (0.83)	No (0.36)
ACE1 (ng/mL) in total group (*n* = 122)	1 vs. 21	24.9 (19.6; 41.9)	Yes (0.029)	No (0.012)	Yes (0.009)
ACE1 (ng/mL) in subgroup (*n* = 23)	1 vs. 21	18.0 (10.5; 47.0)	Yes (0.188)	No (0.846)	No (0.257)
CRP (mg/dL) in total group (*n* = 122)	1 vs. 7	−0.035 (−0.290; −0.015)	No (0.74)	No (0.12)	No (0.77)
	1 vs. 14	0.000 (−0.275; 0.020)	No (0.61)	No (0.29)	No (0.48)
	1 vs. 21	−0.010 (−0.335; 0.055)	No (0.30)	No (0.55)	No (0.47)
CRP (mg/dL) in subgroup (*n* = 23)	1 vs. 7	−0.020 (−3.950; 0.665)	No (0.91)	No (0.75)	No (0.63)
	1 vs. 14	−0.380 (−6.175; −0.115)	No (0.20)	No (0.96)	No (0.21)
	1 vs. 21	−0.160 (−6.185; −0.035)	No (0.17)	No (0.60)	No (0.47)

* The “Difference Median” column refers to the median of the observed differences between the values of the subjects’ biomarkers at a given date and the baseline (e.g., the median of the observed differences between the platelet count on days D14 and D01).

The 95% confidence intervals were calculated using the Wilcoxon signed rank statistic. Total group = 122 patients; Subgroup = 23 patients with progression of the infection (CT Scan); LCLT, L carnitine L-tartrate.

### 3.4. Subgroup analyses in cohort 2

**Figure 4:**
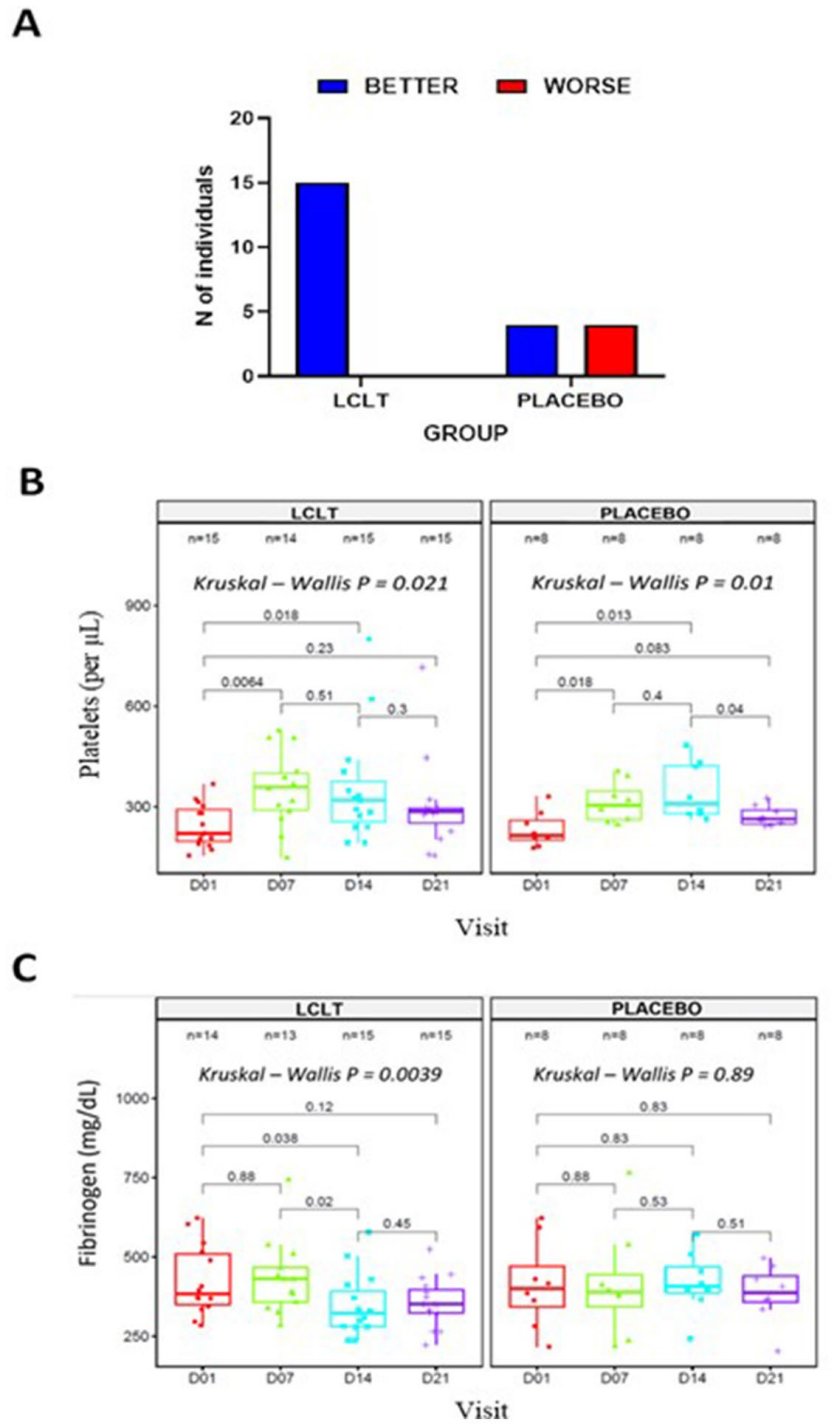
Subgroup analysis in cohort 2. **(A)** Number of participants showing changes in lung lesion on CT imaging from day 7 to 14. Blood samples collected at days 01, 07, 14, and 21 were used to evaluate platelet counts **(B)**; Friedman test, *X*^2^ (1) = 4, *p* = = 0.046, *n* = 4 and Fibrinogen levels **(C)**; Friedman test, *X*^2^ (1) = 4, *p* = = 0.046, *n* = 4. LCLT, L-carnitine L-tartrate..

Following the CT scan findings, we undertook a *post-hoc* analysis, wherein a single radiologist blinded to the study group allocation read the individual CT scan images from all the study time points for all the participants who underwent CT scan. An arbitrary visual images sequence was used to read the current and previous visit images (i.e., day 7 vs. 1, or day 14 vs. 7). Classification criteria were the number of patients showing (1) improvement in pneumonia lesions, (2) worsening of pneumonia lesions, (3) stable pneumonia lesions, and (4) resolution of pneumonia lesions. In this analysis, a subgroup of 23 participants who showed differences in the progression of the infection (represented by <25% of the consolidated lesion images) was identified. This subgroup had worsening lung lesions from day 1 to 7 (Figure 4A). On analysis of image changes from day 7 to 14, there was a significant difference between the LCLT group and the placebo group. The LCLT group images showed significant lesion improvement from day 7 to 14 compared with those of the placebo group. The use of AI deep learning lesion classification confirmed this finding. Our statistical analysis found that a greater number of LCLT group images showed lung lesion reduction compared with those of the placebo group (*p* = 0.006).

Previously evaluated biomarkers were also analyzed for the subgroup of 23 participants. The platelet counts were significantly increased from days 1 and 7 in the LCLT and placebo groups compared to the placebo (*p* = 0.006, Figure 4B). A similar pattern was observed for the LCLT and placebo groups when comparing days 1 and 14 (*p* = 0.018, *p* = 0.013, respectively). The LCLT group had a slight decrease (but not significant) in platelet count from day 7 to day 14, whereas the placebo group did not have a similar decrease. The LCLT group had significantly lower fibrinogen levels on day 14 than those on day 1 (*p* = 0.038) and decreased fibrinogen levels from day 7 to day 14 (*p* = 0.02; Figure 4C). Such decreases were not observed in the fibrinogen levels of the placebo group. No significant differences in CRP levels were observed between the groups (Table 3).

## 4. Discussion

L-carnitine has been suggested to have beneficial effects on COVID-19 due to its anti-inflammatory and anticoagulation effects as well as regulatory role in fatty acid metabolism. Here, we evaluated the potential benefits of L-carnitine supplementation in patients with COVID-19 disease. Our results showed that LCLT supplementation was safe and well tolerated, and may be protective, since we found a decrease in coagulation disturbance markers, as well as reduction in the severity of lung alterations when given to patients with mild COVID-19 (Cohort 2).

Based on a previous study showing the effects of LCLT as a possible inhibitor of SARS-CoV-2 infection due to ACE-1 inhibition (12), we also sought here to evaluate the preventive effects of LCLT supplementations on COVID-19 negative volunteers which living on households with COVID-19-infected people (Cohort 1). However, due to the low infectivity rate circulating at the time in the areas in which this study was performed, only two participants in each group (LCLT or placebo) presented with a positive RT-PCR test for COVID-19. Although CRP levels were significantly increased in the placebo, but not in the LCLT group, due to the low infectivity rate in the Cohort 1 we cannot correlate with disease progression. Therefore, we could not conclude if LCLT supplementation can reduce the rate of infection by SARS-CoV-2.

Given that there were no SAEs reported for participants of either cohort, LCLT appears to be well-tolerated in individuals exposed to or infected with SARS-CoV-2. Although our primary endpoints of SARS-CoV-2 infection rates in Cohort 1 and disease progression in Cohort 2 were unmet, our analysis of the secondary endpoints suggests that LCLT supplementation modulates coagulation via increased platelet count and decreased fibrinogen levels. Also, clinical improvement among patients with SARS-CoV-2 infection was observed between days 7 and 14, at which lung effects are typically appreciated (20).

The significant increase in platelet levels from day 1 to 7 in the Cohort 2 LCLT group suggests that LCLT may have an antithrombotic effect, which could be beneficial for COVID-19 patients with a high rate of thrombosis-induced complications. The fibrinogen level decrease observed in the Cohort 2 LCLT group reflects previous reporting of decreased plasma fibrinogen levels in hemodialysis patients who received LC supplementation (21). These results suggest a trend of normalizing fibrinogen levels and platelet counts in the LCLT group, particularly within the 23-participant subgroup of pneumonia participants.

No significant differences were observed with respect to the percentage of lung lesions when the entire study population of cohort 2 was analyzed. However, a sub-group analysis of 23 subjects showed striking difference in the lung CT scan images between the LCLT and placebo groups. There was more progression on the ground glass opacities (GGOs), consolidations and crazy-paving pattern with bilateral and multifocal distribution in the placebo group compared to LCLT, which suggest LCLT may provide some protection against COVID-19 pneumonia. This indicates that LCLT administration may contribute to a better evolution of COVID-19 patients.

Talebi et al. (22) showed recently that L-carnitine supplementation promoted improvements in several parameters, including O2 saturation, erythrocyte sedimentation rate (ESR), C-reactive protein (CRP), alkaline phosphatase (ALP) activity, lactate dehydrogenase (LDH), and serum creatine phosphokinase (CPK) when compared to the intervention group. Additionally, the authors found no mortality in the L-carnitine group, while 14% of the patients in the control group died. Moreover, Li et al. (23) showed a possible correlation between higher endogenous carnitine levels and decreased susceptibility and better outcome in COVID-19. Altogether, these data corroborate the beneficial effects of L-carnitine in preventing aggravation of COVID-19 seen in our study.

Regarding the levels of serum ACE1, we observed a slight increase in this marker only in Cohort 2 LCLT group comparing days 1 and 21. When calculating ACE1/ACE2 ratio, we did not see any significant difference between the two groups. This is an intriguing finding given that we had observed decreases in ACE2 receptor levels in response to supplementation with LCLT in previous human study (16). The potential reason for this disconnects would be attributed to the fact that the study subjects in the previous study were healthy subjects (16). Nonetheless, more studies are warranted to further elucidate the mechanism by which LCLT may impact SARS-CoV-2 pathogenicity.

In conclusion, our data suggest that LCLT supplementation may have coagulation modulating effects and result in clinical improvement in patients with COVID-19. While the results of this study are promising, future studies, with a larger sample size, are warranted to further explore and validate the impact of L-Carnitine on COVID-19.

## Data availability statement

The raw data supporting the conclusions of this article will be made available by the authors, without undue reservation.

## Ethics statement

The studies involving human participants were reviewed and approved by Ethics Committee of SENAI CIMATEC. The patients/participants provided their written informed consent to participate in this study.

## Clinical trial group members

Afrânio Ferreira Evangelista, Camila Oliveira Valente, Carolina Thé Macedo, Cassio Santana Meira, Crislaine Gomes da Silva, Danielle Devequi Gomes Nunes, Emanuelle de Souza Santos, Katharine Valéria Saraiva Hodel, Leticia de Alencar Pereira Rodrigues, Patrícia Kauanna Fonseca Damasceno, Rodrigo Souza Conceição, and Vinícius Pinto Costa Rocha (Institute of Health Technologies ITS—SENAI CIMATEC). Medical doctors that evalauted the patients and subjects included into the study: Amanda Viana, Suillan pedreira, Carolina Macedo, Célia Brito, Allison Silva, Rayza Silva, Ana Fernandes, Cristiane Queiroz, Cristiane Cunha, Nayra Mascarenhas e Clarissa Souza.

## Author contributions

SD, ZS, KJ, and RD: conceptualization. RB, BM, and MS: methodology. JB, VS, MT, and LA: software. RB, CA, SD, ZS, KJ, and RD: validation. RB, JB, CA, MS, VS, MT, SD, ZS, KJ, and RD: formal analysis. RB, JB, CA, and MS: investigation. JB, BM, and LA: resources. RB, MS, SD, ZS, KJ, and RD: data curation. RB, JB, MS, SD, ZS, KJ, and RD: writing—original draft preparation and writing—review and editing. SD, ZS, and RB: supervision. ZS and JB: project administration. All authors contributed to the article and approved the submitted version.

## Funding

This research received funding support from Lonza Greenwood LLC., Greenwood, SC, United States, and Senai-Cimatec University research and innovation center of Salvador, Bahia, Brazil.

## Conflict of interest

The authors from CIMATEC University Center, who were independent of Lonza Greenwood LLC., conducted the research and have no conflict of interest, which means they have not received shares of Lonza’s stock or participation in any other aspects of Lonza’s business. SD, RD, and ZS are employees of Lonza, while KJ is a consultant to Lonza.

The authors declare that this study received funding from Lonza Greenwood LLC. The funder was involved in the study design and writing of the article. The funder was not involved in the data collection, analysis or data interpretation.

## Publisher’s note

All claims expressed in this article are solely those of the authors and do not necessarily represent those of their affiliated organizations, or those of the publisher, the editors and the reviewers. Any product that may be evaluated in this article, or claim that may be made by its manufacturer, is not guaranteed or endorsed by the publisher.
